# The Long Pentraxin PTX3 as a Humoral Innate Immunity Functional Player and Biomarker of Infections and Sepsis

**DOI:** 10.3389/fimmu.2019.00794

**Published:** 2019-04-12

**Authors:** Rémi Porte, Sadaf Davoudian, Fatemeh Asgari, Raffaella Parente, Alberto Mantovani, Cecilia Garlanda, Barbara Bottazzi

**Affiliations:** ^1^Department of Inflammation and Immunology, Humanitas Clinical and Research Center-IRCCS, Milan, Italy; ^2^Department of Biomedical Sciences, Humanitas University, Milan, Italy; ^3^The William Harvey Research Institute, Queen Mary University of London, London, United Kingdom

**Keywords:** pentraxin, pentraxin 3 (PTX3), inflammation, innate immunity, sepsis

## Abstract

The first line of defense in innate immunity is provided by cellular and humoral mediators. Pentraxins are a superfamily of phylogenetically conserved humoral mediators of innate immunity. PTX3, the first long pentraxin identified, is a soluble pattern recognition molecule rapidly produced by several cell types in response to primary pro-inflammatory signals and microbial recognition. PTX3 acts as an important mediator of innate immunity against pathogens of fungal, bacterial and viral origin, and as a regulator of inflammation, by modulating complement activation and cell extravasation, and facilitating pathogen recognition by myeloid cells. In sepsis, PTX3 plasma levels are associated with severity of the condition, patient survival, and response to therapy. In combination with other established biomarkers, PTX3 could improve stratification of sepsis patients and thus, complement the system of classification and monitoring of this disease.

## Introduction

The first line of defense against invading pathogens is provided by the innate immune system that comprises both cellular and humoral arms. In contrast with adaptive immunity, the innate immune receptors are germ-line encoded receptors called pattern recognition molecules (PRMs), which recognize conserved structure on the surface of pathogens, the so-called pathogen-associated molecular patterns (PAMPs). Based on their localization, PRMs have been divided into cell-associated receptors and soluble molecules. Cell-associated PRMs are located in different cellular compartments and include endocytic receptors (e.g., scavenger receptors), signaling receptors such as Toll-like receptors (TLRs), the NOD-like receptors and RIG-like receptors (1). Fluid-phase molecules contribute to the humoral arm of innate immunity, and function as the ancestor of antibodies. They are diverse in terms of structure, expression, and specificity, but share basic functions, including regulation of complement activation, opsonisation of pathogens and apoptotic cells and regulation of inflammation (1). Fluid-phase PRMs, which include complement components, mannose-binding lectin (MBL), surfactant protein, ficolins, and pentraxins (PTXs), are expressed by different cell types including myeloid, epithelial and endothelial cells (1).

Pentraxins are a superfamily of phylogenetically conserved proteins, sharing an ~200 amino acid long domain characterized by the presence of the so-called “pentraxin signature” in their carboxy-terminal, which is a conserved 8 amino acid long-sequence (His-x- Cys-x- Ser/Thr-Trp-x- Ser, where “x” represents any amino acid). Based on the length of the N-terminal region, these multifunctional proteins are divided into short and long pentraxins. Short pentraxins include C-reactive protein (CRP) and serum amyloid P (SAP), whereas PTX3, PTX4, neuronal pentraxin 1 (NP1) and NP2 belong to the long family (2).

The short pentraxins CRP and SAP are ~25-kDa proteins produced by hepatocytes in response to pro-inflammatory signals, such as IL-6, and constitute the main acute phase proteins in human and mouse, respectively. They have a peculiar quaternary structure with five (CRP) or ten (SAP) identical subunits arranged in pentameric symmetry with 51% amino acid sequence identity. In human, plasma level of CRP is low in healthy adults (below 3 mg/L), but it can increase as much as 1,000 folds in 48 h during inflammation. In contrast, SAP plasma level is constitutively 30–50 mg/L even during the acute phase response in human (1).

CRP is the first identified PRM and its name derives from its capacity to recognize C-polysaccharide of *Streptococcus pneumoniae*. The CRP gene is located on chromosome 1q23.2 locus in human. The human SAP gene is located on chromosome 1 and shares with CRP the exon/intron structure (2).

CRP and SAP share functional properties including recognition of pathogens, regulation of complement and interaction with Fcγ receptors (FcγR), resulting in enhancement of pathogen phagocytosis (1).

PTX3 (also named TSG-14) was identified during the early 1990s as a secreted protein containing a C-terminal pentraxin domain (2). The human PTX3 gene is located on chromosome 3q25 and is organized in three exons coding for the leader signal peptide, the long N-terminal domain (amino acids 18–178) and the C-terminal pentraxin domain (amino acids 179–381) (1). In contrast to the C-terminal, the N-terminal region of PTX3 is unrelated to any known protein domain (1). Human and mouse PTX3 display 92% amino-acid conservation and 82% of these amino-acid residues are identical, suggesting a strong evolutionary pressure to maintain both its structure and function.

PTX3 has a complex quaternary structure characterized by two tetramers linked together by covalent bonds to form an octamer of 340 kDa that is the main form of the molecule and shows greater functional activity, comparing with the tetrameric form (3).

Human and murine PTX3 gene promoters are characterized by numerous potential binding sites for inflammatory transcription factors, including PU.1, AP-1, NF-κB, SP1, and NF-IL-6 sites (2).

**Figure 1:**
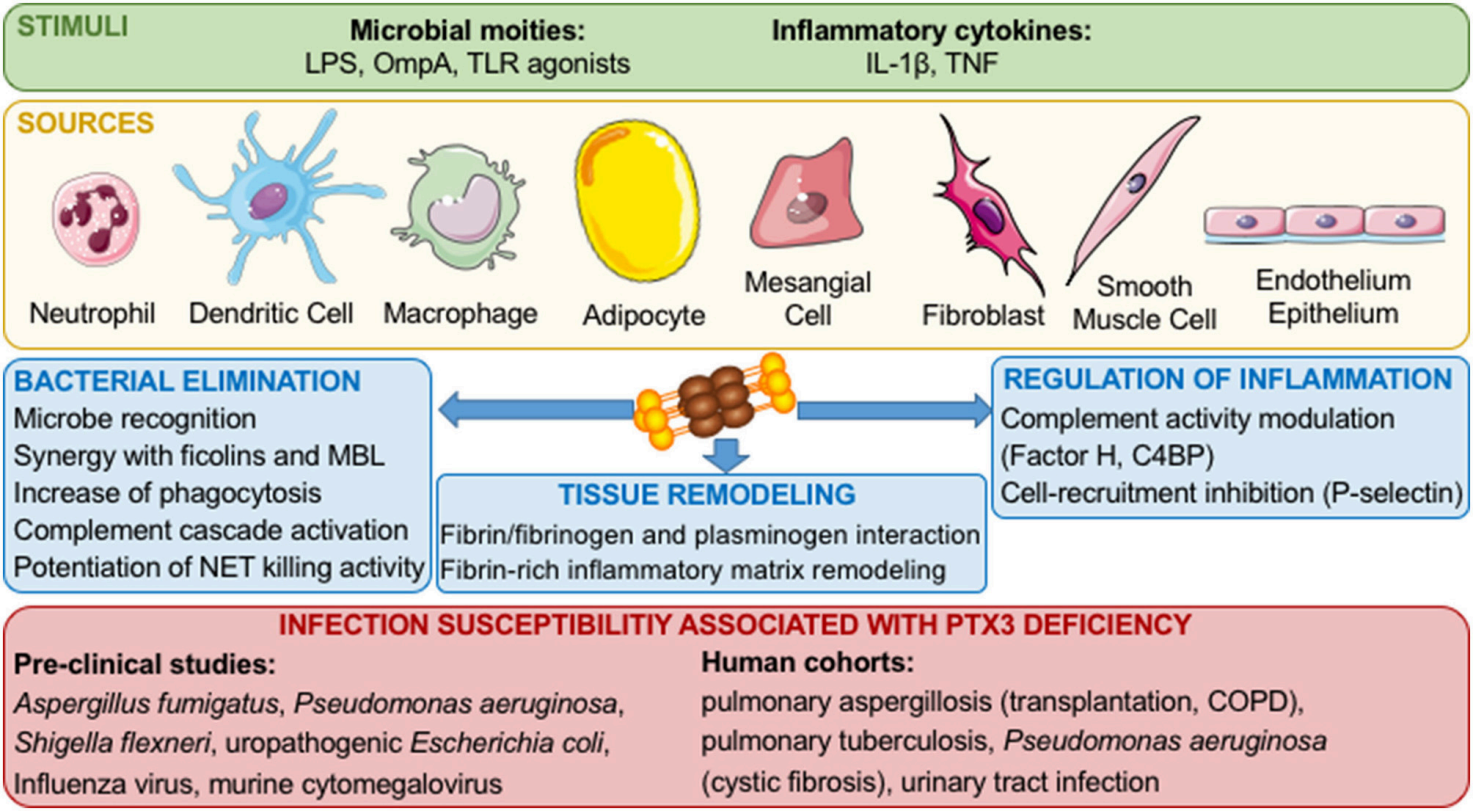
Role of PTX3 in innate immune responses and inflammation. Inflammatory stimuli and cellular sources of PTX3 are shown. Once released, PTX3 interacts with microorganisms, complement components, phagocytosis receptors, P-selectin, and components of the haemostatic system and fibrinolytic cascade, thus promoting pathogen clearance, tuning inflammatory responses and promoting tissue remodeling.

While CRP and SAP are mainly produced by the liver upon stimulation by IL-6, PTX3 is secreted by different cell types, including dendritic cells (DCs), monocytes, macrophages, fibroblasts, synovial cells, chondrocytes, adipocytes, epithelial cells, vascular endothelial cells, smooth muscle cells, mesangial cells, granulosa cells, and glial cells in response to TLR agonists, inflammatory cytokines (such as IL- 1β and TNFα), microbial moieties [LPS or outer membrane protein A (OmpA)], and microorganisms (Figure 1) (1, 4). Finally, neither T and B lymphocytes or NK cells express PTX3 mRNA (2), while PTX3 expression in mature neutrophils is still debated. Jaillon et al. reported that immature neutrophil precursors express PTX3 transcripts and synthesize the protein during differentiation in the bone marrow, while mature peripheral blood neutrophils were not able to express PTX3 mRNA. PTX3 is stored as a reservoir in lactoferrin- positive granules in a ready to use form and is promptly released after microbial recognition and inflammatory signals (5). In contrast with the study by Jaillon et al. (5) and Imamura et al. (6) reported the expression of PTX3 mRNA in freshly isolated mature neutrophils before and after stimulation with LPS (5, 6). In addition, it has been recently reported that splenic B helper neutrophil cells (NBh cells) can express PTX3 transcript in response to GM-CSF and LPS (7). PTX3 produced by NBh cells bound marginal zone B cells and promote IgM to IgG class switch recombination, thus representing a bridge between the humoral arms of the innate and the adaptive immune system.

Based on its quaternary structure, PTX3 interacts with various ligands, exerting different biological activities. In the context of innate immune responses, PTX3 binds different complement components and modulates complement activity (2). In addition, PTX3 binds P-selectin and tunes recruitment of neutrophils limiting inflammation (8). PTX3 also binds to a wide range of microorganisms, including fungi, bacteria, and viruses and is involved in resistance to selected infectious diseases (2). In addition, by interacting with a series of ligands and tuning inflammatory responses, PTX3 has various roles in different settings, such as wound healing and tissue remodeling, cardiovascular diseases, fertility, and cancer (1).

In line with the behavior of CRP as inflammatory biomarker, PTX3 has been developed as a novel marker for infectious or inflammatory disease severity.

Here, we will present the experimental evidence showing the functional roles of PTX3 and its potential as biomarker focusing on infections and sepsis.

## Role of PTX3 in Innate Resistance to Infections

Since its discovery, PTX3 has been described to modulate innate immune mechanisms involved in protection against infectious diseases (Figure 1). Several studies using *Ptx3*-deficient mice showed an increased susceptibility to fungal, bacterial and viral pathogens such has *Aspergillus fumigatus, Pseudomonas aeruginosa, Shigella flexneri*, uropathogenic *Escherichia coli*, Influenza virus and murine cytomegalovirus (9–13). In contrast, a recent study showed a deleterious effect of PTX3 during Ross River virus (RRV) infection in mice (14). In the context of RRV infections, it has been shown that PTX3 increases viral replication and entry in target cells (14). Furthermore, the transgenic overexpression of PTX3 in mice is responsible of resistance against *Klebsiella pneumoniae* respiratory infection and increased phagocytosis of *Paracoccidioides brasiliensis* by macrophages (15, 16). The protective effect of PTX3 has been investigated treating infected animals with the recombinant protein. Indeed, PTX3 treatment alone or in combination with antifungal compounds, showed a protective effect in *A. fumigatus* infections, (17–20). In addition PTX3 administration is protective also against infections with Influenza virus, murine cytomegalovirus, *Neisseria meningitidis*, and *P. aeruginosa* in neonates and during chronic infections (10, 11, 21–24). Interestingly, in addition to being effective against opportunistic bacteria known to be responsible of sepsis (e.g., *K. pneumoniae* and *P. aeruginosa*), PTX3 was shown to have protective effect by reducing the mortality in mouse models of sepsis induced by histone infusion, LPS-induced endotoxemia and cecal ligation and puncture, through its N-terminal domain (25, 26).

### Antimicrobial Mechanisms

Similar to short pentraxins, PTX3 binds a number of selected bacteria, fungi and viruses (2). The capacity of PTX3 to bind microorganisms has been described first with *A. fumigatus* conidia: in this context PTX3 was shown to increase the internalization and killing of conidia by alveolar macrophages (5, 9, 27). This opsonic activity depends on the complement system, since PTX3 enhances neutrophil phagocytic activity in the presence of normal human serum, but this effect is abolished with heat-inactivated serum. Interestingly, the same study demonstrated that the opsonic activity of PTX3 is Ig-independent, since PTX3 enhanced phagocytosis in the presence of Ig-depleted serum (27). In addition, by using complement-depleted sera (Factor B and C3) and reconstituted with purified complement components (C3, Factor B, Factor H, Factor D and Factor I), Moalli et al. showed the main involvement of the alternative complement pathway in the pro-phagocytic activity exerted by PTX3. Finally, with integrin and FcγR blocking antibodies, they demonstrated that FcγRIIa (CD32) and complement receptor 3 (CD11b/CD18) are required for the interaction with PTX3-opsonized *A. fumigatus* conidia, are recruited into the phagocytic cup and cooperate in the phagocytosis process amplified by PTX3 (27).

This opsonic capacity has been extended to others pathogens such as uropathogenic *E. coli* and *P. aeruginosa* (12, 21). PTX3 is also able to bind Influenza virus, murine cytomegalovirus, *P. brasiliensis*, encapsulated *N. meningitidis*. An interaction was described with outer membrane vesicles or selected antigens from *N. meningitidis* or with OmpA of *K. pneumoniae* (10, 11, 15, 24, 28, 29).

The formation of neutrophil extracellular traps (NETs) by neutrophils releasing DNA, histone and bactericidal compounds is an important pathway to control infections. Jaillon et al. observed the localization of PTX3 in human neutrophil granules and the secretion after bacterial and PRR stimulation. In this study they also showed PTX3 localized in NETs formed after neutrophil activation (5). Proteomics analysis revealed that PTX3 forms complexes with two anti-microbial proteins [azurocidin (AZU1) and myeloperoxidase (MPO)] associated to NETs (30). More recently, PTX3 localization in NETs has been confirmed, and the colocalization with AZU1 and MPO has been defined more accurately (31). Further investigation will be needed to understand the involvement of PTX3 interaction with AZU1 and MPO in their antibacterial role during NET formation.

### Regulation of Complement Activation

PTX3 interaction with microorganisms is not restricted to directly increase phagocytosis. PTX3 can be used as a sensor by complement to rapidly recognize microbial patterns and enhance its activation and its antimicrobial activity. Once bound, PTX3 recruits the complement component C1q and activate the classical pathway of the complement cascade, leading to C3 deposition on apoptotic cells (32). In contrast, the interaction between PTX3 and C1q in the fluid phase prevents complement deposition onto apoptotic cells and their clearance by dendritic cells (33). PTX3 also interacts and activates the lectin pathway of complement. Indeed, Ma et al. demonstrated that PTX3 bound on *A. fumigatus* conidia interacts with ficolin-2 and leads to an increase of complement deposition on *A. fumigatus* (34). Later on, this group showed that the interaction of PTX3 with ficolin-1 on the cell surface of apoptotic cells leads to an increase of their phagocytosis by human monocyte–derived macrophages, but this does not occur for *A. fumigatus* conidia (35). Ma and co-workers also described the interaction of PTX3 bound to *Candida albicans* with mannose-binding lectin (MBL), and the consequent enhancement of C3 and C4 deposition on the yeast and its phagocytosis by neutrophils (36).

In addition to the ability to enhance complement activation, PTX3 has been described to interact with complement regulators. Deban et al. described the interaction of PTX3 with factor H (FH), the main regulator of the alternative complement pathway. The interaction between apoptotic cell surface bound-PTX3 with FH enhanced iC3b deposition on the cells, thus preventing the cleavage of C3b and its deposition on the apoptotic cells (37, 38). Moreover, another study described the direct interaction of PTX3 with C4-binding protein (C4BP), another regulator of complement activation. In this study, the authors showed that PTX3 recruits C4BP on late apoptotic cells determining C4b cleavage and reduction of the formation of the final complement lytic complex C5b-9 (39). Thus, these studies demonstrate that PTX3 can recruit the two complement regulators FH and C4BP to limit excessive complement activation.

### Modulation of the Inflammatory Response

The development of the inflammatory response and the control of inflammation during infections need to be tightly regulated. PTX3 has been described to control cell recruitment at inflamed sites by binding P-selectin (8). In their study, Deban et al. observed that *Ptx3*-deficiency is associated with an increase of leukocyte rolling interactions and suggested that PTX3 binding to P-selectin would limit leukocyte rolling and thus inflammatory cell infiltration. The involvement of P-selectin has been confirmed in a model of post-ischemic renal injury. In this model, *Ptx3*-deficient mice exhibit a higher leukocyte recruitment compared to wild type mice that is inhibited by a P-selectin blocking antibody (40). More recently, in a model of chemical carcinogenesis, the lack of PTX3 has been associated with an exacerbated cancer-related inflammation explained by a defect of complement regulation, as indicated by excessive C3 deposition and a higher amount of the highly inflammatory compound C5a in *Ptx3*-deficient compared to —competent mice (41) In murine models, until now, only one study has associated the alteration of inflammatory control by PTX3 with the susceptibility to *K. pneumoniae* infections (16). In this study, PTX3 was shown to regulate the production of TNFα and nitric oxide, and depending on the intensity of the inflammatory response induced by a given inoculum, promote or inhibit neutrophil influx and the susceptibility to pulmonary infection with *K. pneumoniae*.

### Orchestration of Tissue Repair

Recent *in vitro* and *in vivo* studies demonstrated that PTX3 interacts with components of the haemostatic system and fibrinolytic cascade, in particular with fibrinogen/fibrin and plasminogen, at acidic conditions, which occur in damaged tissues. This interaction promoted remodeling of the fibrin-rich inflammatory matrix ensuring a normal tissue repair in different experiment models (42). The interaction between PTX3, fibrin and plasminogen is potentially relevant during specific infections or in septic conditions, but further studies are needed to address this aspect.

## PTX3 in Human Infections

The homology with CRP, that was first discovered in the serum of patients with acute pneumococcal pneumonia and is upregulated in the context of inflammation and infections, prompted investigations on the involvement of PTX3 as biomarker of infections. In inflammatory or infectious diseases, PTX3 behaves as an acute phase response protein and the basal blood levels observed in normal condition (25 ng/ml in the mouse and <2 ng/ml in human) rapidly increase during the course of pathological conditions, reaching in humans 100–1,000 ng/ml, depending on the severity (43). Cardiovascular diseases, cancer and infections, all characterized by an inflammatory origin, are the most relevant pathologies showing upregulated plasma PTX3 levels (43–48). In general, PTX3 increase over basal levels could be already appreciated beginning from 6–8 h after insult, while CRP needs longer times and levels start to be significantly altered only after 24–30 h. This is likely the result of local production of PTX3 following detection of bacterial moieties or tissue damage by innate immune cells, combined with the production of the primary pro-inflammatory cytokines IL-1β and TNFα, main inducers of PTX3. By contrast CRP is systemically produced by the liver in response to IL-6, with a process that requires longer time.

Focusing on the context of infective diseases, PTX3 has been characterized as a biomarker of severity and outcomes in different infections caused by bacteria, fungi or viruses (Table 1). Patients with pulmonary aspergillosis, tuberculosis, dengue virus infection, meningococcal disease leptospirosis and shigellosis have increased PTX3 plasma levels that correlate with disease severity and could act as predictor of unfavorable outcomes (13, 49–51, 59, 61). PTX3 levels in plasma and bronchoalveolar lavage fluids (BALF) discriminate patients with invasive aspergillosis from those with other conditions, including lung cancer, community-acquired pneumonia (CAP), pulmonary cryptococcosis and *Aspergillus fumigatus* colonization (62, 63). Circulating levels of PTX3 were associated with SOFA (sequential organ failure assessment) score and case fatality in patients with bacteremia caused by *Staphylococcus aureus, Streptococcus pneumoniae*, β-hemolytic streptococcae or *Escherichia coli* (53).

**Table 1:** Clinical studies reporting the use of PTX3 as biomarker of infectious diseases or sepsis/shock.

	Pathology^*^	**Main results**	**References**
Bacterial infections	• *Mycobacterium tuberculosis* infection • Leptospirosis • Meningococcal disease • Shigellosis • Community acquired pneumonia • Pyelonephritis • Bacteremic patients • Pneumonia in intubated patients • Bacterial infections in COPD^a^ • Febrile patients • Parapneumonic effusion	Correlation with disease severity & hospital stay; predictor of bloodstream infections, response to treatment & mortality; biomarker of bacteria-associated exacerbation in COPD; early indicator of shock.	(49) (50) (51) (52) (53) (54) (55) (56) (12) (57) (58) (13)
Viral infections	• *Dengue* virus • Lower respiratory tract infections (mostly RSV^c^, IV^d^ and hRhV^e^)	Correlation with disease severity	(59) (60)
Fungal infections	• *Aspergillus fumigatus* (proven/probable)	Monitoring of fungal infection	(61) (62) (63)
SIRS, sepsis or septic shock		Correlation with organ dysfunction & disease severity; predictor of mortality; associated to amputation in NSTI^b^ patients and with lower APGAR scores in preterm neonates.	(64) (65) (66) (67) (68) (69) (70) (71) (72) (73) (74) (75)
Infections/sepsis in acute decompensated cirrhosis		Predictor of disease severity and case fatality	(76)
Cardiogenic shock		Predictor of mortality	(77)

a COPD, Chronic Obstructive Pulmonary Disease.

b NSTI, Necrotizing Soft Tissue Infections.

c RSV, Respiratory Syncytial Virus.

d IV, Influenza Virus.

e hRhV, human rhinovirus.

* *Studies focused on microbial infections are reported in the bacterial, fungal or viral infection groups. Studies on sepsis/shock irrespective to the causing agent(s) are reported in the SIRS, sepsis or septic shock group*.

PTX3 was shown to predict bloodstream infection and severe disease in febrile patients admitted to emergency departments (52), indicated acute respiratory distress syndrome (ARDS) in critically ill patients (78), and correlated with pneumonia severity and length of hospital stay in adults with CAP (54). Serum and urinary PTX3 levels are increased in pyelonephritis patients and correlated with parameters of disease severity (12). In patients with chronic obstructive pulmonary disease (COPD), levels of PTX3 in sputum samples are associated with bacterial infections and can potentially be a marker of exacerbation of the disease (58). On the opposite, in children with lower respiratory tract infections, mostly of viral origin, PTX3 levels were increased irrespective of the causative agent, but were indeed correlating with the febrile peak and reflected disease severity (60).

The diagnostic accuracy of PTX3 as local marker of infection has been strengthened by the observation that its levels can identify microbiologically confirmed pneumonia in BALF and plasma of mechanically ventilated patients (55, 57). The accuracy of PTX3 was compared to other biomarkers used to follow critically ill patients, namely procalcitonin (PCT) (79, 80) and soluble triggering receptor expressed on myeloid cells 1 (sTREM-1). PCT, released by thyroid C cells in healthy subjects, is induced in multiple tissues by LPS and proinflammatory cytokines during infections. Circulating levels of PCT are a reliable biomarker of bacterial infection and provide a guide to follow antibiotic therapy in critical ill patients with suspected infections (81). sTREM-1 is expressed by myeloid cells in BALF and is useful to make diagnosis of pneumonia infection in intubated patients (82). Area under the curve-receiver operating characteristics (AUC-ROC) analysis indicated that the diagnostic accuracy of PTX3 in BALF was better than the other biomarkers currently used, namely PCT, CRP, and sTREM-1. More in general, PTX3 levels in pleural fluids can discriminate parapneumonic exudative effusion of infectious origin from malignant or other cause pleural effusion (56).

Monitoring the level of PTX3 has not only been described to be a good biomarker for detecting infections, but also to follow the response to therapy. Indeed, in a population of 220 newly diagnosed patients with *Mycobacterium tuberculosis* infections, PTX3 plasma levels were significantly decreased in individuals who responded to therapy compared to patients with treatment failure (49). After antibiotic treatment PTX3 levels were reduced in plasma of patients with CAP (54), or in urine of pyelonephritis patients (12).

### Impact of Genetic Polymorphisms

In human, PTX3 is not only interesting for its biomarker features. Indeed, its deficiency, as described in the preclinical studies above, has been associated with an increased susceptibility to infections. In a first cohort of patients undergoing hematopoietic stem-cell transplantation, three single-nucleotide polymorphisms (SNPs) within the *PTX3* gene were identified that were consistently associated with a defect in PTX3 expression in BALF, lung-biopsy specimens, and innate immune cells (83, 84). The defective expression of PTX3 is mainly observed in neutrophils and was proposed to be due to messenger RNA instability, but the exact mechanism is presently unknown. For these patients, genetic variants of the *PTX3* gene of transplant donors were associated with the susceptibility to invasive aspergillosis (IA). Several studies confirmed the association of these genetic polymorphisms to pulmonary aspergillosis and other fungal infections in hematopoietic stem cell transplantation and solid organ transplantation, in particular lung, (83–87). One study has also shown a significant association between PTX3 SNPs and the susceptibility to pulmonary aspergillosis in patients with Chronic Obstructive Pulmonary Disease (COPD) (88). Clinical studies also support the relevance of the preclinical models. Indeed, PTX3 SNPs have been associated with increased susceptibility to pulmonary tuberculosis in West Africa patients, *P. aeruginosa* infections in cystic fibrosis Caucasian patient and urinary tract infection in Swedish patients (12, 89, 90).

The higher susceptibility to IA associated with a particular haplotype of PTX3 that is likely related to lower production of the protein, further supports the non-redundant role of PTX3 in host defense against *A. fumigatus* and opens up the prospective for a therapeutic use of this long pentraxin in infections.

## PTX3 as Biomarker in Sepsis

A deregulated host response to infections results in a systemic inflammatory response that activates a cascade of events known as systemic inflammatory response syndrome (SIRS), eventually leading to sepsis. The induction of PTX3 by primary pro-inflammatory cytokines and microbial components prompted to analyse the circulating levels of this protein in sepsis and its complications. In a small cohort of critically ill patients, Mueller and co-workers observed increased PTX3 plasma levels compared to healthy control donors, with a gradient from SIRS to sepsis and septic shock, and a significant correlation with disease severity as assessed by clinical scores (64). Most important, PTX3 levels on admission or day 2 were significantly associated to mortality.

Since this first observation, several papers analyzing PTX3 plasma levels in patients with sepsis and its complications were published (see Table 1 for a summary). In a group of critically ill patients, persistently high PTX3 levels from the first days after diagnosis were significantly associated with poor outcome. Levels of PTX3 were correlated with disease severity, organ dysfunction and markers of coagulation activation and, when compared with other biomarkers (i.e., IL-6, TNFα and CRP) showed a stronger correlation with clinical parameters (65). Bastrup-Birk et al. analyzed PTX3 levels in patients admitted to ICU for SIRS and showed a significant correlation with Simplified Acute Physiology Score 2 (SAPS2). In this group of patients, Cox regression analysis revealed a significant association between PTX3 levels and 90-day mortality, in contrast with CRP levels (66). In a group of 112 patients admitted to the ICU with septic shock, baseline PTX3 levels were the only independent risk factor for 28-day mortality, in contrast with CRP and PCT (72). Similarly, in a group of more than 500 patients admitted to emergency room with suspected infection, PTX3 levels predicted severe sepsis and mortality while CRP was not associated to case fatality (67). In patients with necrotizing soft tissue infections, PTX3 plasma levels were associated with amputation and were higher in individuals with sepsis (69). PTX3 was compared with PCT and CRP in a small group of patients undergoing early goal-directed therapy and initial resuscitation (73). In this cohort, PTX3 was the only biomarker significantly different between survivors and non-survivors in all the time points considered (day 0, 3, and 7). Despite a correlation with PCT or CRP, plasma PTX3 measured at day 0 was the only independent marker of 28-day all-cause mortality. PTX3 levels were consistently higher in patients with sepsis or septic shock and were predictor of mortality also when evaluated according to the latest Sepsis-3 definition (70, 74, 91). In addition, PTX3 correlated with disease severity and degree of organ dysfunction. Finally, a study in a small group of preterm infants showed a correlation between PTX3 levels in newborns and overall worsen neonatal outcome (i.e., lower APGAR score, elevated respiratory distress syndrome rate, clinical sepsis, and prolonged NICU stay) (68).

Severe systemic inflammation, organ failure and septic shock are major life-threatening complications in different pathological conditions. A significant percentage of cirrhotic patients develops bacterial infections that can trigger systemic inflammation, organ failure and septic shock. Circulating levels of PTX3 are increased in these patients, compared to well-compensated cirrhotic patients, and predict disease severity and risk of mortality (76). Similarly, plasma PTX3 levels are increased in patients with cardiogenic shock and predict 3-month mortality, but levels over the time course are not associated to the presence of infections (77).

A major limitation of the studies mentioned above is the heterogeneity and limited numbers of patients enrolled. To overcome this point, PTX3 was recently measured in a large group of patients enrolled in a biomarker substudy of the Albumin Italian Outcome Sepsis (ALBIOS) trial, a multicentre open-label randomized controlled trial that enrolled patients with severe sepsis or septic shock [NCT00707122] (92). Within the total population of 1,818 patients enrolled, PTX3 levels were measured in 958 patients at day 1, 2, and 7 after ICU admission. Results obtained in this large cohort demonstrated that PTX3 levels on day 1 were higher compared to levels in healthy population and were correlated with severity. In addition, PTX3 levels on day 1 predicted development of novel organ dysfunctions, including cardiovascular and renal dysfunction (71). In each time point analyzed, PTX3 levels were consistently higher in patients who died than in survivors, and levels on day 7 showed a significant predicting value of 90-day mortality. Similarly, slower decreases in PTX3 levels from day 1 to day 2 were independently associated with higher mortality (71). In addition, this study showed that PTX3 levels at day 1 were inversely associated with platelets count and predicted coagulation dysfunctions. These results are in line with results obtained in preclinical studies, showing a link between PTX3 and haemostasis and fibrinolysis (42), and suggest that PTX3 could be a useful marker to stratify patients with coagulation dysfunctions. In conclusion, this study confirmed in a large and controlled population that PTX3 levels are highly increased in severe sepsis and even more in septic shock, and that impaired normalization or reduction of PTX3 levels in the first days predicts multiorgan dysfunction and risk of mortality.

The latter study, and many others, showed that PTX3 concentration dropped immediately following effective treatment during ICU stay, suggesting that monitoring PTX3 levels could be useful to follow responsiveness to therapy (67, 70–72).

**Figure 2:**
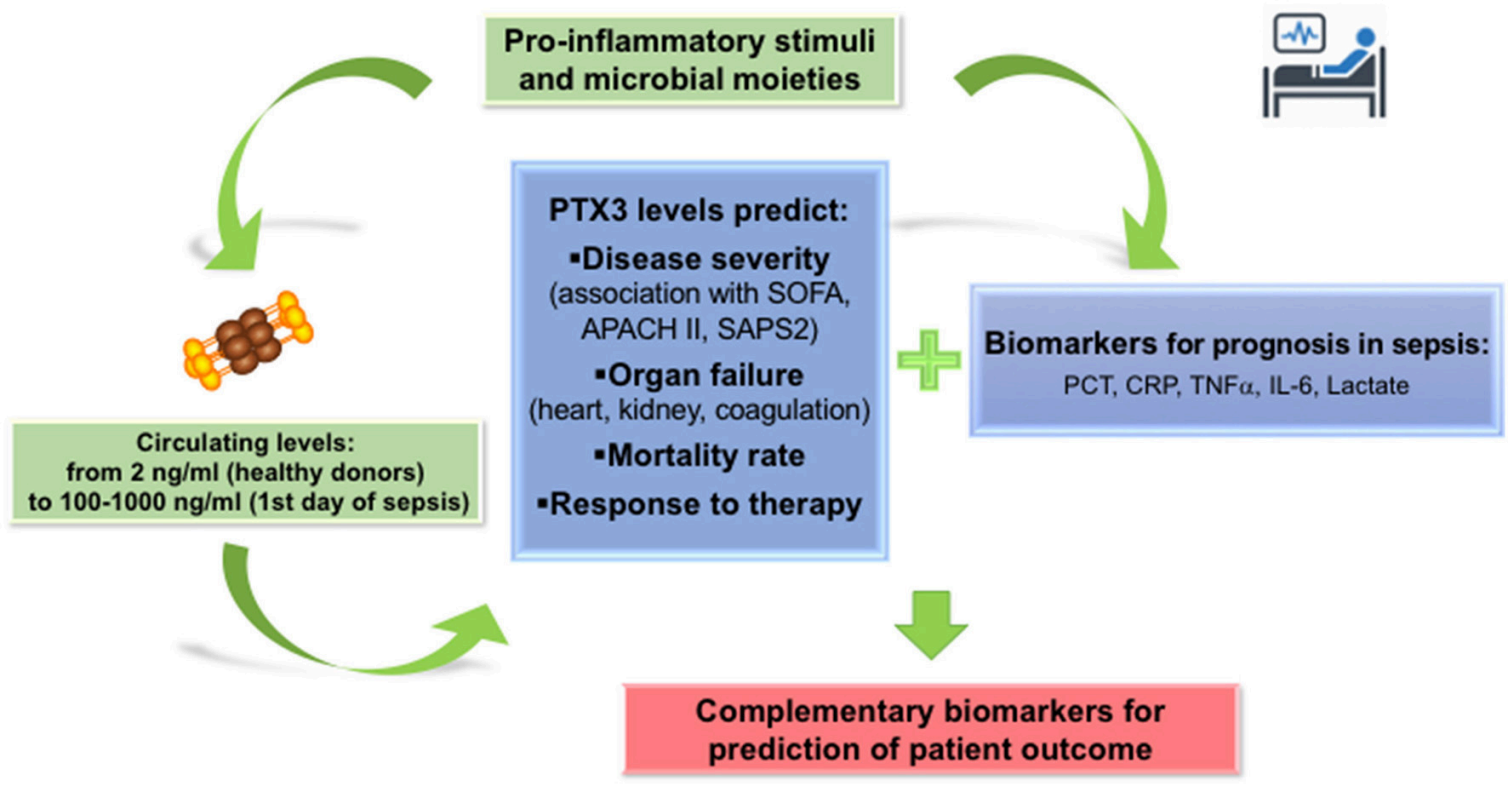
PTX3 in the prognosis of sepsis. PTX3 can be considered as a reliable biomarker for prognosis of sepsis patients, predicting severity of disease, incidence of new organ failures and mortality risk. Addition of PTX3 levels measured on the first day in ICU or ER to the panel of markers routinely used in Intensive care units and emergency room units, may increase the accuracy of prognosis in sepsis patients.

While there is a general consensus on the increase of PTX3 plasma levels in septic patients, and the correlation with severity from SIRS to sepsis and septic shock, the diagnostic superiority of PTX3 over other biomarkers, such as PCT, IL-6, CRP and lactate, is still under debate. A possible strategy to increase the diagnostic accuracy is to analyse simultaneously a combination of different biomarkers (Figure 2). In a prospective analysis, the changes in plasma levels of PTX3, PCT and lactate during the first week in ICU stay were compared to severity scores of sepsis (SOFA and APACHE II). All the biomarkers correlated with SOFA and APACHE II and were associated with 28-day mortality by univariate and multivariate Cox regression analysis (75). A model combining these three biomarkers improved significantly mortality prediction of patients with sepsis (75). Similarly, PTX3 in combination with IL-6 improved the risk stratification of patients with sepsis or septic shock as classified using the updated sepsis-3 definition (70).

### Limitation

Physiological concentration of plasma PTX3 are influenced by several factors, including sex, age, pregnancy, triglyceride levels and body mass index (93, 94), as well as by PTX3 allelic variants (83, 95), which induce variation of PTX3 levels in the range of about 1 ng/ml. In contrast, during severe infections and sepsis PTX3 levels pass from 1–2 ng/ml to 100–600 ng/ml, suggesting that variations due to these factors are not a major limitation for the use of PTX3 as a biomarker in sepsis. However, several pathological conditions, such as cardiovascular diseases and kidney diseases (96–98) induce increased PTX3 circulating levels and must be taken into consideration as potential confounding factors in the stratification of septic patients with these underlying conditions. Along the same line, pregnancy disorders, such as intrauterine growth restriction (94), lead to increased PTX3 levels in neonates and may be confounding factors in neonatal sepsis (68).

## Concluding Remarks

Sepsis and its complications are a major cause of death in ICU, with a mortality rate still ranging from 10% in the systemic inflammatory response syndrome (SIRS), to 60% in septic shock, while the short term mortality remains around 20% (99–101). At the moment validated diagnostic tests are not available, thus diagnosis is still largely based on clinical evaluation of the patient (91). Identification of reliable circulating biomarkers that can predict severity and mortality of disease, improve risk stratification and help in defining optimal treatment, could be thus highly valuable in the clinical practice. So far various biomarkers have been investigated, but none of them has appropriate specificity or sensitivity to be routinely applied in the clinical practice (99, 102).

PTX3 is a soluble pattern recognition receptor rapidly produced in response to primary pro-inflammatory signals and microbial recognition. Data collected over the years demonstrated that circulating PTX3 levels increase rapidly in response to infections and play important regulatory roles on inflammation, regulating complement activation, cell extravasation and pathogen recognition by myeloid cells. In sepsis and septic shock, PTX3 discriminates from healthy controls, and non-survivors consistently show levels higher than survivors. In addition, PTX3 levels at early time points and/or a lower decrease in response to first therapies are an independent predictor of mortality. Several recent data demonstrate that PTX3, in combination with other established biomarkers, could be useful to improve stratification of patients with sepsis or septic shock. Thus, PTX3 could complement the system of classification of the disease, contributing to define a group of useful biomarkers and strengthening their use in the diagnosis and monitoring of sepsis and septic shock.

## Author Contributions

RéP, SD, FA, and RaP did the literature search and wrote the manuscript. AM, CG, and BB critically revised the manuscript and approved the final version.

### Conflict of Interest Statement

The authors declare that the research was conducted in the absence of any commercial or financial relationships that could be construed as a potential conflict of interest. The reviewer SA declared a shared affiliation, though no other collaboration, with one of the authors, SD, to the handling editor.
